# Challenges in radiopharmaceutical importation across six Southern African Development Community countries: an exploratory study

**DOI:** 10.3389/fmed.2026.1786884

**Published:** 2026-03-06

**Authors:** Vukosi G. Mkhombo, Lerato Mosima, Beverley Summers

**Affiliations:** Radiopharmacy Unit, Department of Pharmaceutical Sciences, School of Pharmacy, Sefako Makgatho Health Sciences University, Pretoria, South Africa

**Keywords:** importation, nuclear medicine, radiopharmaceuticals, radiopharmacy, SADC, supply chain

## Abstract

**Introduction:**

Radiopharmaceuticals are the backbone of nuclear medicine, essential for accurate diagnosis and effective treatment of various diseases, particularly cancer. Yet across the African region, patients still face delays in nuclear medicine services because radiopharmaceuticals are not available when needed. Due to radionuclide short half-lives, supply delays compromise their effectiveness because of rapid radioactive decay.

**Aim:**

This study aimed to investigate, in six English-speaking South African Development Community (SADC) countries, factors that affect radiopharmaceutical importation, identify similarities and differences in their importation processes, determine the customs handling procedure, and identify areas for potential improvement in their importation.

**Method:**

The study was an exploratory pilot study. An online questionnaire SurveyMonkey® was distributed to healthcare professionals and distributors with experience in radiopharmaceutical importation in the six target countries via email.

**Results:**

Based on 10 completed and two partially completed surveys. The preliminary results suggest that importation costs, frequent logistical delays due to limited transport options, inconsistent customs clearance procedures, and a limited number of suppliers negatively affect access to radiopharmaceuticals in the region. These factors collectively disrupt timely delivery and compromise the availability of nuclear medicine services.

**Discussion:**

The importation of radiopharmaceuticals between individual SADC member states is hindered by high import costs, unharmonized customs regulations, limited production capacity, and inefficient transport systems. South Africa remains the main regional supplier, yet its market dominance and high costs restrict access for neighboring countries. Strengthening regional collaboration, harmonizing customs procedures, and expanding local production capacity are essential to improve the accessibility and sustainability of radiopharmaceuticals in the region.

## Introduction

Developing countries are woefully underserved with nuclear medicine services, and this is certainly the case in sub-Saharan Africa (1, 2). Even operational centers face budgetary and procurement challenges. Hence, when radiopharmaceuticals are actually ordered and paid for, their timely arrival is critical for maximal utilization. The production and supply of radionuclides rely on complex and highly specialized infrastructure, including research reactors, cyclotrons, and radionuclide generators (3). These production pathways are often geographically distant from end-users, creating significant time and logistical constraints for Nuclear Medicine Departments dependent on imported radiopharmaceuticals (4). The distance between production facilities and user centers complicates access and availability, particularly for radionuclides with short half-lives, with potential consequences for diagnostic accuracy and therapeutic effectiveness (5).

Following production, radiopharmaceuticals must be labeled, packaged, stored, and transported in strict accordance with national regulations, which are generally aligned with international standards for the safe handling of radioactive materials (6). Transportation may occur by road, rail, air, or sea and requires reliable, time-efficient logistics due to the rapid decay of radionuclides, often measured in hours (5). Delays or inefficiencies in transport can significantly reduce available activity at the point of use, thereby compromising clinical outcomes (4).

In Africa, access to radiopharmaceuticals remains highly unequal due to the limited production capacity, dependence on a few supply centers, and complex logistics. This scarcity restricts timely diagnosis and treatment, particularly for cancer, creating major disparities in patient access (7, 8). The situation is especially challenging in African countries, where reliance on single sources increases vulnerability to supply chain disruptions (7). Furthermore, the complex shipping and logistics associated with radiopharmaceuticals pose additional barriers, especially in developing nations and countries like those in the Southern African Development Community (SADC), a regional economic bloc comprising 16 Member states in Southern Africa that aims to promote trade integration and cooperation (9).

Despite the growing demand for radiopharmaceuticals, supply chain challenges remain poorly documented in Africa (10). Regional trade agreements and diverse customs procedures create additional complexity for importers (9). A Kenyan study (10) highlighted challenges to the supply chain of radiopharmaceuticals, which were predominantly delays in customs clearance at entry ports. No similar study has been conducted in the SADC region.

Ensuring the safe handling of radiopharmaceuticals is very important, with particular emphasis on transportation, requiring collaboration among neighboring states (11). Several countries within the SADC have nuclear medicine centers. South Africa, as one of the most developed countries in the region, has 17 public nuclear medicine centers and 92 private nuclear medicine centers. Other countries within the SADC region have far fewer centers, and they are less extensively equipped, thus relying more on the importation of radiopharmaceuticals (12).

Our study presents a pilot assessment aimed at investigating the factors that affect the importation of radiopharmaceuticals in six English-speaking SADC countries, namely, South Africa, Tanzania, Namibia, Mauritius, Zimbabwe, and Zambia, as these are the only English-speaking countries in the region with operational nuclear medicine centers. Focusing on these countries ensured linguistic consistency in data collection and allowed for comparison within a shared regional development framework. Identifying and addressing these factors is crucial in improving healthcare delivery and enhancing patient care by improving the availability and accessibility of radiopharmaceuticals.

## Materials and methods

### Study design

The study employed an exploratory pilot approach, to acquire more information on the challenges experienced when importing radiopharmaceuticals in the SADC region. This will then set the basis for future extensive research on the subject. Six English-speaking SADC countries were included, as these were the only countries in the region with operational nuclear medicine centers at the time of the study.

### Data collection

Data was collected between March and August 2025 using an online questionnaire generated with SurveyMonkey® and distributed via email to healthcare professionals and distributors with experience in radiopharmaceutical importation. Follow-up reminder emails were also sent weekly to participants from countries where responses had not yet been received or completed. The questionnaire was available in English only to ensure linguistic consistency across countries and reduce potential translation errors, given resource constraints for professional translation.

Participants were recruited using purposive and snowball sampling. This approach may introduce selection bias and limit generalizability, as participants were not randomly selected, but it was appropriate given the limited target population. Self-reporting bias is also possible and was addressed by comparing participants’ responses with existing literature and documented practices.

### Data entry and analysis

Responses from SurveyMonkey were exported to Microsoft Word and Excel for organization and review.

For the descriptive quantitative data, the summary statistics generated by SurveyMonkey were utilized to describe the patterns and trends of responses across the six English-speaking SADC countries.

All the qualitative data responses were manually reviewed to identify recurring ideas and key issues. Similar responses were grouped into categories and compared across countries to highlight both common and country-specific factors.

The categorized findings were subsequently interpreted in relation to the study objectives, providing a structural basis for the presentation of results. Given the pilot nature of the study, no inferential statistical analysis was undertaken. The findings provide descriptive insights into radiopharmaceutical importation challenges and can inform the design of future studies where inferential analysis may be appropriate.

## Results

The results obtained from the respondents are presented in detail below.

### Participation

A target sample size of 23 participants was set for this study. Invitations to participate in the study were sent to 23 participants using a snowballing approach and referrals. A purposive sampling strategy was used to recruit respondents. Due to the limited number of facilities in the region, the achievable sample was small. A total of 12 Participants from six English-speaking SADC countries responded to the survey, yielding a response rate of 52%. However, only 10 participants (43% response) completed the questionnaire in full, and their responses were included in the analysis. However, this was considered adequate for a pilot, exploratory assessment.

The 10 respondents included distributors, nuclear medicine technologists, and radiopharmacists with direct involvement in the importation, handling, and use of radiopharmaceuticals. The countries represented in the study were: Namibia, Tanzania, Mauritius, Zimbabwe, Zambia, and South Africa (SA). Participation demographics are summarized in Table 1.

**Table 1:** Participation summary and participants’ demographics.

Country	Study population	Sample size (*n*)	Responses (*n*)	Occupation
Namibia	4	4	2	2 Nuclear Medicine technologists
Tanzania	2	2	2	2 Radiopharmacist
Mauritius	2	2	1	1 Radiopharmacist
Zimbabwe	1	1	1	1 Radiopharmacist
Zambia	1	1	1	1 Radiopharmacist
South Africa	13	13	5	2 Radiopharmacists and 3 distributors
Total	23	23	12	

### To identify the similarities and differences in the importation process of radiopharmaceuticals used

#### Radiopharmaceuticals imported and their country of origin

Respondents provided information on the diagnostic and therapeutic radiopharmaceuticals they import, the cold kits used in their facilities, and the countries of origin for these products. Where multiple participants responded from the same country (e.g., Namibia and South Africa), their answers were presented separately due to notable differences in the type of radiopharmaceuticals and their source countries. The tables below summarize the diagnostic radiopharmaceuticals, therapeutic radiopharmaceuticals, and cold kits imported by each respondent, along with their respective countries of origin.

Table 2 shows the imported diagnostic and therapy radionuclides across the participating facilities in six countries. Almost all (85.71%) countries outside SA that participated in the study procure their ^131^I primarily from SA except for one facility in Tanzania, which procures it from Turkey.

**Table 2:** Diagnostic and therapy radionuclides imported by the six SADC countries (*n* = 10).

Country	Diagnostic radionuclides	Therapy radionuclides
Namibia *n* = 2	R1: ^99m^Tc (SA) ^131^I, ^123^I, [^131^I] I-MIBG (Not stated)	[^131^I] I-MIBG [^131^I] I-NaI (SA)
	R2: ^99m^Tc (SA and Poland)	[^131^I] I-MIBG [^131^I] I-NaI (SA)
Tanzania *n* = 2	R1:^99m^Tc (SA and Morocco)	[^131^I] I-NaI (SA)
	R2: ^99m^Tc (SA and Turkey) ^131^I (Turkey)	^131^I (Turkey)
Zambia *n* = 1	^99m^Tc (SA)	[^131I^] I-NaI (SA)
Zimbabwe *n* = 1	^99m^Tc (SA)	[^131^I] I-NaI (SA)
Mauritius *n* = 1	^99m^Tc (Poland transit through SA) ^18^F (Reunion Island) ^131^I (Poland)	[^131^I] I-NaI (SA)
South Africa *n* = 3	R1: ^99m^Tc (Netherlands) ^68^Ga (Germany)	[^177^Lu]Lu-Dotatate/toc (Germany), [^177^Lu]Lu-PSMA (Germany), [^90^Y]Y-citrate (Netherlands), [^90^Y]Y-microsphere (Europe), [^90^Y]Y-SirSpheres (Europe), [^131^I]I-MIBG (Poland and Budapest), [^181^Re]Re-HEDP (Netherlands), [^153^Sm]Sm-EDTMP (Netherlands), [^225^Ac]Ac-PSMA (Russia and Germany), [^169^Er]Er-Citrate (Netherlands), [^161^Tb]Tb-PSMA and DOTATATE (Netherlands)
	R2: ^99m^Tc (Curium, UK), ^177^Lu, ^203^Pb (Germany, USA and Isreal)	^203^Pb (USA)
	R3: ^99m^Tc (Germany)	[^225^Ac] Ac-PSMA (Russia)

Diagnostic radionuclides such as ^99m^Tc are widely imported across all countries, with SA, Tanzania, and Namibia sourcing from multiple suppliers, including SA, Poland, Morocco, and Turkey.

Table 3 shows the imported cold kits across the participating facilities in six countries. Radiopharmaceutical cold kits for ^99m^Tc-based procedures are consistently used across all countries. SA is the only country in the region that utilizes a wider variety of radiopharmaceutical cold kits, including specialized kits such as PSMA and Vasculocis®. It is also the only country that imports a vast range of therapeutic radionuclides from different countries across the globe, mainly from European countries and a few from Russia and the United States. This data highlights significant variation in radiopharmaceutical diversity and access across the region, with South African facilities having the most extensive inventory and variety compared to other SADC countries.

**Table 3:** Cold kits imported by the six SADC countries.

Country	Cold kits
Namibia *n* = 2	R1: DISIDA, DMSA, DTPA, MAA, MAG3, MDP, MIBI, Nanocolloid, HMPAO, Mebrofenin, RBC, Tin colloid, pyrophosphate (SA and Poland) Tektroytyd®, Leukoscan® (SA)
	R:2 DISIDA, DMSA, DTPA, MAA, MAG3, MDP, MIBI, Nanocolloid, RBC, Tin colloid, Tektroytyd, Pyrophosphate, Leukoscan® (SA and Europe)
Tanzania *n* = 2	R:1 DISIDA, DMSA, DTPA, MAG3, MDP, MIBI, Mebrofenin (SA and Morocco)
	R:2 DMSA, DTPA, MAG3, MDP, MIBI (SA and Poland)
Zambia *n* = 1	MDP (SA)
Zimbabwe *n* = 1	DISIDA, DMSA, DTPA, HMPAO, MAA, MAG3, MDP, MIBI (SA)
Mauritius *n* = 1	DMSA, DTPA, MAA, MAG3, MDP, MIBI, RBC, Nanocolloid (Poland)
South Africa *n* = 3	R:1 DISIDA, DMSA, DTPA, HMPAO, MAA, MAG3, MDP, Mebrofenin, MIBI, Nanocolloid, RBC, Pyrophosphate (Hungary, Poland, and Netherlands) PSMA (Poland, Germany, and Mexico), Tin colloid and Tektroytyd® (Poland), Leukoscan®, Vasculocis® (Netherlands)

#### Supplier selection criteria

**Figure 1:**
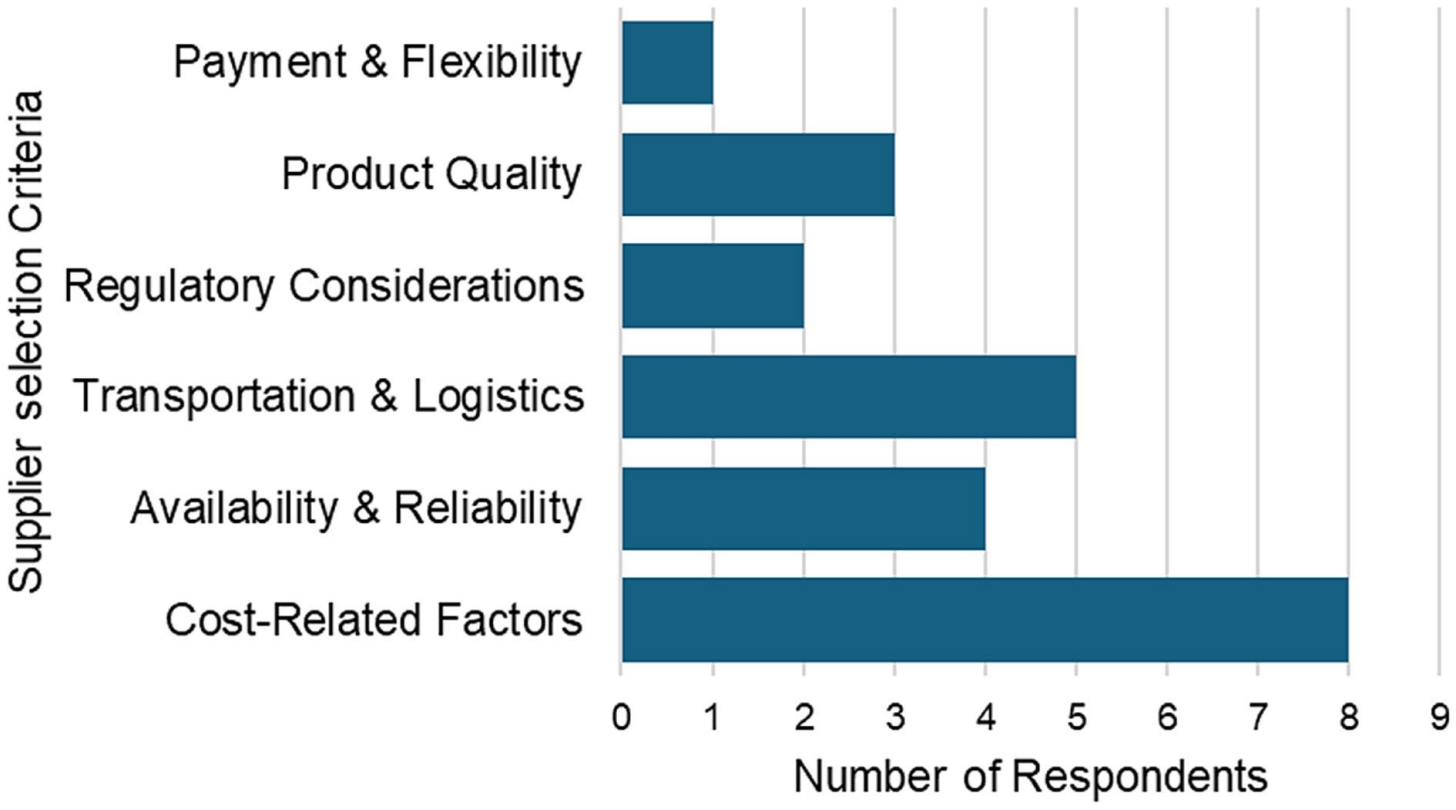
Factors influencing supplier selection for radiopharmaceuticals (*n* = 10).

Respondents identified the following six major categories as factors they consider when selecting a supplier: cost, availability, certification, product quality, technical stability, and payment flexibility (Figure 1).

##### Cost considerations

Predictably, cost emerged as the most frequently mentioned factor. Respondents emphasized both the price of the product and the cost of importation. One participant from South Africa compared the financial implications of importing versus producing in-house, suggesting that local production could offer cost advantages. Currency fluctuations and payment in foreign currencies, e.g., USD, EUR, ZAR, were also highlighted as challenges, especially in countries with strict currency regulations.

##### Availability and reliability

The availability of radiopharmaceuticals and the reliability of the supplier in delivering them on time were also key concerns. Participants stressed the importance of having a consistent supply of products, particularly for radionuclides with short half-lives, where timely delivery is critical.

##### Transport and logistics

Transport factors were consistently mentioned, particularly the speed of delivery, cost of transportation, and the distance between the supplier and the facilities. Given the time-sensitive nature of radiopharmaceuticals, especially those with short half-lives, delays in transportation were seen as a major barrier. One respondent also pointed out that some shipping carriers refuse to transport radiopharmaceuticals, limiting delivery options and increasing access limitations.

##### Regulatory considerations

Several participants indicated that national importation policies and customs regulations significantly impact supplier selection. In addition, Good Manufacturing Practice (GMP) certification was cited as a criterion for choosing suppliers.

##### Product quality

Factors such as the half-life, stability of the product during transportation, and the technical quality of the kits were also mentioned. Participants expressed the need for high-quality, stable radiopharmaceuticals that retain efficacy upon arrival.

##### Payment and financial flexibility

Lastly, the flexibility of payment terms and the supplier’s responsiveness in resolving payment or delivery issues were also considered important. Suppliers who can accommodate different payment methods and respond to operational challenges promptly were preferred.

Figure 1 shows how many participants cited each of the above-mentioned factors. As shown in Figure 1, cost-related factors were the most frequently cited considerations when selecting a radiopharmaceutical supplier (*n* = 8). Transport and logistics (*n* = 5) and availability and reliability of supply (*n* = 4) were also reported as important factors. Fewer respondents emphasized product quality (*n* = 3), regulatory considerations (*n* = 2), and payment flexibility (*n* = 1). These findings indicate that while cost is the primary driver of supplier selection, logistical reliability and consistent inventory are also critical concerns for facilities in the region.

#### Lead time from order to delivery

Respondents provided estimates of the typical lead time between placing an order for radiopharmaceuticals and receiving them. Reported lead times varied across countries and participants. In South Africa, one respondent noted that the lead time depends on the product, while the other two reported approximately 1 week for ^99^Mo/^99m^Tc generator and 14–20 days for other radiopharmaceuticals. In Namibia, respondents indicated 2–3 days when ordering from South Africa and 2–4 weeks from other countries. The respondent from Mauritius reported a lead time of approximately 1 month. In Tanzania, the reported times were 7 days and 2 weeks. Zambia and Zimbabwe each reported lead times of around 7 days.

### Mode of transport

**Figure 2:**
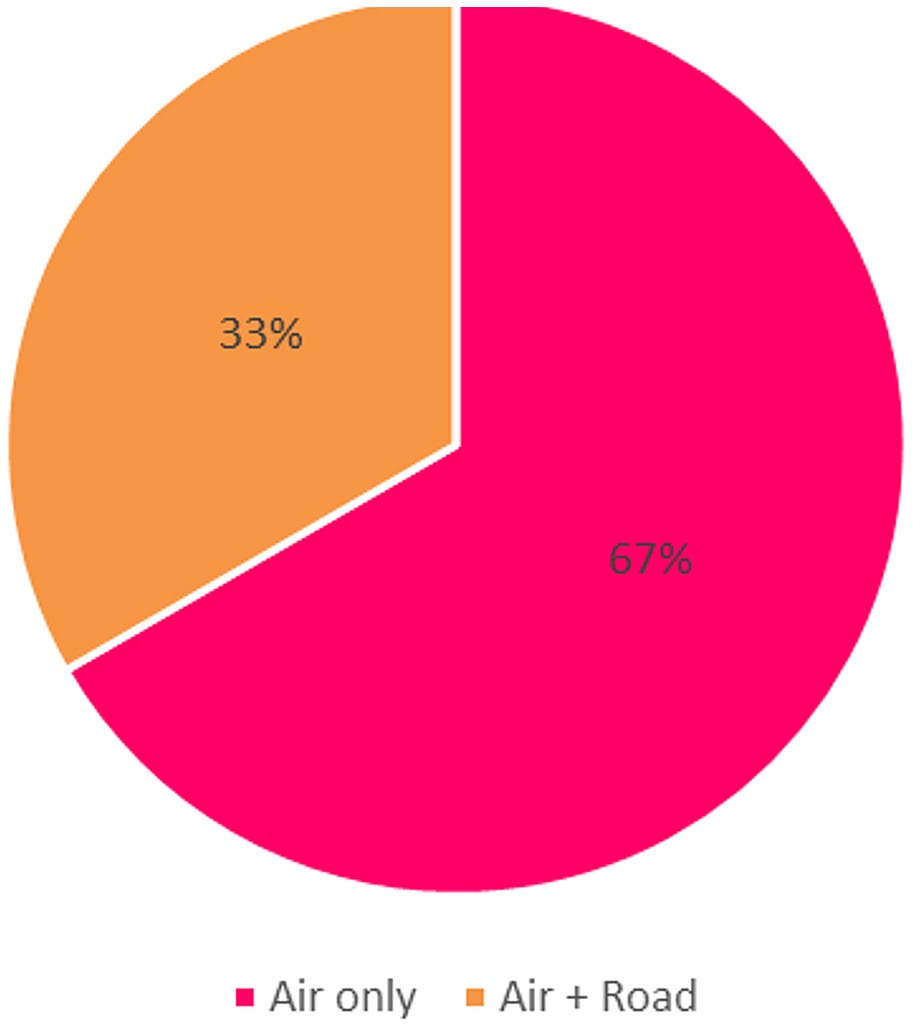
Mode of transport for radiopharmaceutical imports.

Respondents described the transport methods by which radiopharmaceuticals are imported into their countries. The majority reported using air transportation for international shipments, reflecting the time-sensitive nature of radiopharmaceuticals (Figure 2). In Tanzania, Zimbabwe, Zambia, and Mauritius, radiopharmaceuticals are primarily imported via air transport. Respondents from SA and Namibia reported the use of both air and road transport (Figure 2).

The results, as shown in Figure 2, revealed that air transport is the predominant mode of delivery for radiopharmaceuticals. Two-thirds of respondents (67%) reported relying exclusively on-air transport, while one-third (33%) indicated using a combination of air and road transport.

#### Notification of radiopharmaceutical arrival

Respondents indicated various sources of notification regarding the arrival of radiopharmaceutical shipments at the airport. Respondents could select multiple sources of notification. The notification sources reported were:

- Namibia: Customs authorities and courier service.
- Tanzania: Courier service only.
- Zambia: Sender only.
- Zimbabwe: Customs authorities and sender.
- Mauritius: Courier service only.
- South Africa: Customs authorities, courier service, and sender.

### To determine the customs handling process of radiopharmaceuticals

#### Exemption from customs duties and/or taxes

Responses to the question on whether customs duties on radiopharmaceuticals are exempted or not varied, with only two countries (Tanzania and Zimbabwe) in this region having customs duties exemptions, while other countries (SA, Mauritius, Zambia, Namibia, Tanzania) are not exempted from paying customs duties. This indicates misalignment of regulatory requirements among SADC countries.

#### Required documentation for customs clearance

To clear radiopharmaceuticals through customs, respondents highlighted a range of required documents. The documents required are summarized in Table 4.

**Table 4:** Documents required by customs to clear radiopharmaceuticals.

Document type	Namibia *n* = 2	Tanzania *n* = 2	Zambia *n* = 1	Zimbabwe *n* = 1	Mauritius 1	South Africa *n* = 3
Invoice (10)	✓✓	✓✓	✓	✓	✓	✓✓✓
Import permit (9)	✓✓	✓✓	✓	✓	✓	✓✓
Certificate of Origin (6)	✓	✓		✓	✓	✓✓
Bill of lading (8)	✓	✓✓	✓	✓	✓	✓✓
Other documents: Airway Bill (AWB), Dangerous Goods Declaration (DGD), S21 Approval (1)						✓
License from the Tanzania Atomic Energy Commission (1)		✓				

Each tick mark (✓) represents one respondent who indicated that the specified document was required to clear radiopharmaceuticals.

Across all countries, invoices and import permits were the two consistently mentioned documents. Some countries require additional documentation, such as the Certificate of Origin, Bill of Lading, and various regulatory approvals, depending on national requirements.

#### Awareness of customs agents on the nature of radiopharmaceuticals

Variation in customs awareness and processes was observed among countries, as summarized in Table 5.

**Table 5:** Summary of the custom agents’ awareness of the nature of radiopharmaceuticals.

Country	Awareness of agents	Education plan/notes
Namibia	Partially	Not all agents are aware, and no systems are in place for training
Tanzania	Partially	Some awareness, uncertain if systems are in place for training
Zambia	Partially	Training systems are underway
Zimbabwe	Yes	Agents are aware
Mauritius	No	Agents are unaware; no educational system in place or underway
South Africa	Yes	Experienced agents are used to handle radioactive materials

Knowledge about radiopharmaceuticals among customs agents varies between countries. While SA and Zimbabwe reported high awareness, other countries highlighted gaps in knowledge and a lack of formal training systems for customs personnel involved in handling radiopharmaceuticals.

#### Delays in customs clearance and their consequences

Delays in customs clearance of radiopharmaceuticals were reported by participants in South Africa, Zambia, Namibia, Tanzania, and Mauritius.

In SA, delays resulted in postponement or cancelation of treatments and production, depending on the radionuclide’s half-life and product’s shelf life. Others noted that radiopharmaceuticals could arrive decayed, out of calibration, or with insufficient activity, resulting in fewer patients being served.

In Zambia, delays sometimes required the rescheduling of patients’ appointments. In cases involving iodine therapy capsules, reduced activity due to decay meant some patients received doses below the prescribed level.

In Namibia, delays led to the rescheduling of patients, particularly when radiopharmaceuticals were received over weekends. Participants also reported that in some cases, radioactive decay rendered radiopharmaceuticals unusable.

In Tanzania, respondents mentioned that reduced activity at delivery affected both the dose and the number of patients who could be served on the given schedule.

In Mauritius, delays disrupted daily workflow, leading to the rescheduling of procedures planned for the day.

Zimbabwe was the only country where no customs-related delays were reported.

#### From the port of entry to the facility

The responses vary greatly and depend largely on the proximity of the airport to the nuclear medicine facility. The responses are detailed below.

Namibia: Deliveries are completed by road, as the airport is approximately 40 km from the facility.

Tanzania: Delivery is done either by air or road, although air is noted as the easiest and preferred method.

Zambia: A courier company appointed by the Minister of Health is responsible for delivering the radiopharmaceuticals to the receiving facility.

Zimbabwe: The facility arranges its own transport to collect the radiopharmaceuticals directly.

Mauritius: Radiopharmaceuticals are loaded [at the airport] onto a van or lorry and transported directly to the facility.

South Africa: Radiopharmaceuticals are delivered by a radiopharmaceutical company or a licensed transport or courier company familiar with handling radioactive products. These delivery companies are often connected to the nominated clearing agency.

### To investigate factors affecting the importation of radiopharmaceuticals

#### Country-to-country transport challenges

Respondents were asked whether there were challenges when transporting radiopharmaceuticals from countries of origin. Six participants reported no challenges, while a few indicated challenges:

Both SA and Namibia reported challenges related to limited international flights and high import costs. In SA, one respondent noted that while there are no embargoes or major restrictions, limited flights and very high costs were concerns. Similarly, respondents in Namibia highlighted high importation costs and difficulties with flight availability, including cancelations and lack of clearance to carry radioactive materials.

In Mauritius, the respondent noted that flight delays due to extreme weather and strict regulations in supplier countries presented barriers. It was also reported that the radiopharmaceuticals are not always recognized as fully fledged pharmaceuticals, which affects international handling.

#### Port-to-facility transport challenges

Respondents were asked whether there were challenges when transporting radiopharmaceuticals from the port to their nuclear medicine facilities. Eight out of 10 participants reported no challenges, while two respondents from Tanzania reported difficulties. The respondents explained that:

Some courier companies refuse to transport radiopharmaceuticals due to a lack of knowledge and licensing requirements.

Couriers must be licensed by the Tanzanian Atomic Energy Commission to transport radiopharmaceuticals, and delays in issuing these licenses contribute to transport delays.

Flight cancelations sometimes result in product loss due to the short half-life of radiopharmaceuticals.

One respondent noted unreliable transport between Dar-es-Salaam and Mwanza, with most airlines refusing to carry radioisotopes.

Delays in customs clearance and logistics contribute to shortages of radiopharmaceuticals in facilities.

A respondent from Mauritius reported no challenges but commented that starting January 2025, the Radiation Protection Authority requires a Safety Assessment Report for transporting radiopharmaceuticals, indicating new regulatory requirements may affect transport processes.

#### Cost of customs duties and taxes

When asked whether the cost of customs duties and taxes presents a challenge in the importation of radiopharmaceuticals, nine out of 10 respondents reported that it is not a challenge. However, one respondent from SA highlighted that while duties themselves are not problematic, delays in the customs clearance process increase overall costs.

#### Other country-specific challenges

Respondents from some countries reported unique challenges that were not widely shared across the region. These challenges are specific to their local context.

##### South Africa

Cost of shielding: One respondent cited the cost of shielding as a challenge.

Limited importers and market monopoly: Another cited that there are few companies involved in the importation of radiopharmaceuticals, giving them the power to monopolize the market and control prices to their advantage.

##### Tanzania

Delays in customs clearance: One respondent reported that customs processes delay the clearance of radiopharmaceuticals.

Price increases-it was noted that the price of both the nuclides and pharmaceuticals has increased significantly, particularly those sourced from SA.

Lack of government support-law and policy makers have little or no knowledge about radiopharmaceuticals, government priorities, and other synthetic drugs, resulting in unfriendly laws and low availability.

Importation of the radiopharmaceutical generator and cold kits is expensive, and there is no local production, making radiopharmaceuticals unaffordable for many patients.

##### Namibia

Transport costs: A respondent noted that transport costs were a challenge but manageable when road transport is minimized.

Customs delays: Radiopharmaceuticals get stuck or delayed at customs because all the imported products must be cleared.

##### Mauritius

Sourcing difficulties are due to the small market size.

**Figure 3:**
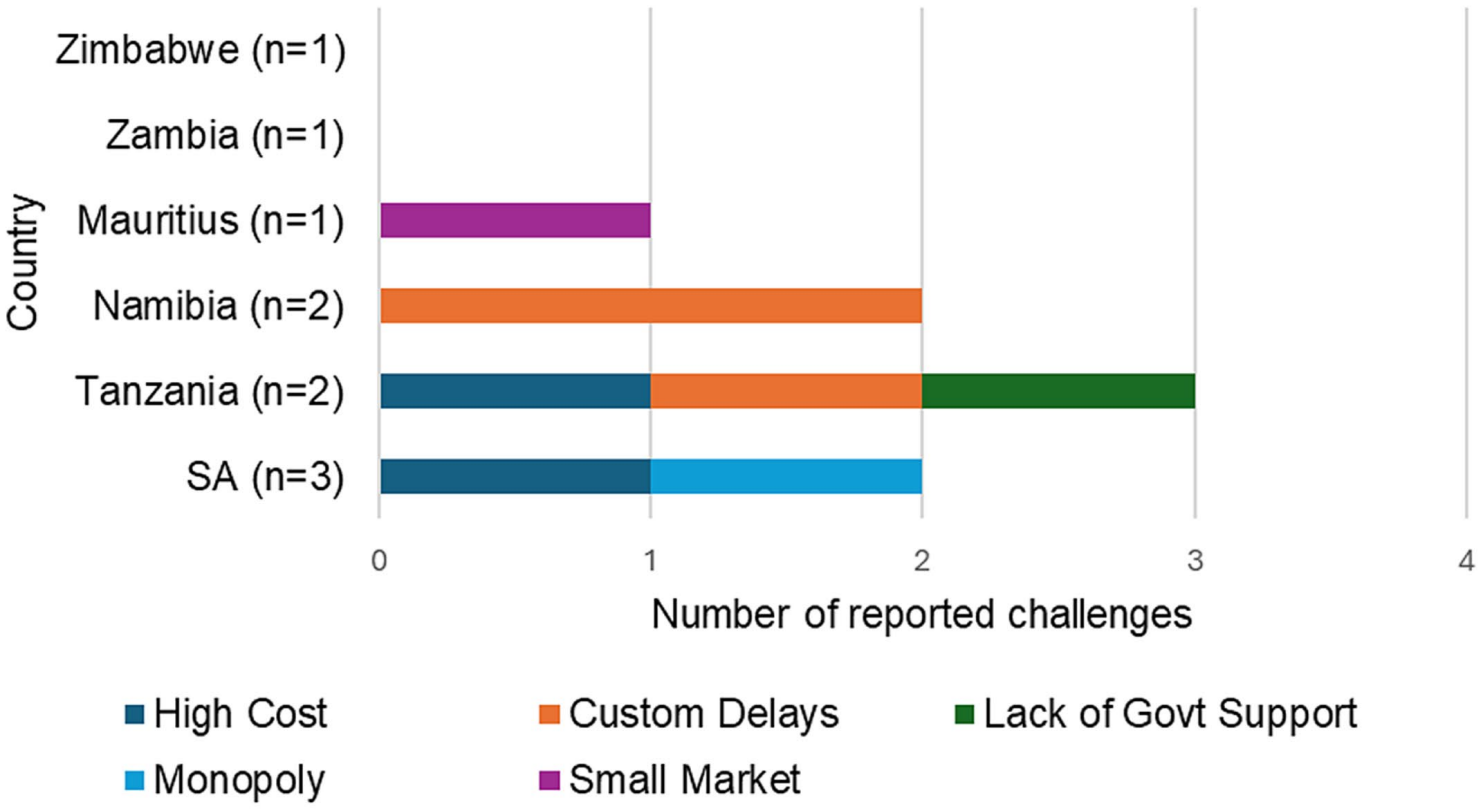
Summary of country-specific importation barriers which had special mention.

No challenges reported: Some respondents from Zimbabwe, Zambia, and one from SA reported no significant challenges in the importation of radiopharmaceuticals, as seen in Figure 3.

### To identify areas for potential improvement in the importation of radiopharmaceuticals

#### Areas of potential improvement

Respondents’ recommendations focused on enhancing government support, improving infrastructure, optimizing import and distribution processes, and increasing local production capacity. Table 6 summarizes the country-specific challenges and the corresponding improvements proposed by the respondents.

**Table 6:** Challenges and the recommended improvement for country-specific challenges.

Challenge	Recommended improvement
Custom clearance delays	Educate customs staff about radioisotopes and radiopharmaceuticals, emphasizing how slow clearance (up to a day) affects patients and nuclear medicine departments.
High prices and lack of local production	Develop more research reactors and research facilities in Africa to produce radionuclides and test pharmaceuticals locally, through collaboration between universities, government, and the private sector. Open local centers for cold kit production to ensure timely availability and low costs, and reduce reliance on expensive imports.
Poor government support	Increase government support to prioritize availability and accessibility by allocating funds/subsidies, covering investigation costs through National Health Insurance, prioritizing radiopharmaceuticals in policies, and engaging in partnerships with other countries or organizations.
Small market	Implement pooled procurement.
Limited importers and market monopoly	Approve more centers licensed to import and distribute radiopharmaceuticals.

One respondent recommended the creation of real-time order systems and real-time tracking to improve efficiency and reduce supply delays. In SA, one respondent also noted that only ^131^I, ^68^Ga, and ^177^Lu are currently produced locally, with other radiopharmaceuticals requiring importation. From Tanzania, a respondent highlighted that while SA has almost all the cold kits needed, they are extremely expensive. By contrast, Turkey offers cheaper cold kits but with a limited variety. The respondent suggested that SA review its price policy to enable neighboring countries to place orders.

## Discussion

The findings highlight a strong dependence on South Africa (SA) as a regional radiopharmaceutical supplier, consistent with evidence that it is the only African country among English-speaking African countries with commercial-scale radiopharmaceutical production capacity in the region. It also has the largest number of nuclear medicine facilities (over 100) (2, 12, 13). This production capability enables both the utilization and distribution of a wider range of radiopharmaceuticals.

The widespread reliance on ^99m^Tc and ^131^I underscores their importance in clinical nuclear medicine, with ^99m^Tc remaining as the cornerstone of diagnostic nuclear medicine and ^131^I the principal radionuclide for thyroid imaging and therapy (14, 15). However, the reliance on imports leaves most SADC countries vulnerable to supply delays and radionuclide decay during transport, particularly for those sourcing from Poland, Turkey, and Morocco (13, 16).

Cold kit usage also revealed disparities. While standard kits for ^99m^Tc-based procedures were available across the region, SA’s access to specialized kits such as PSMA and Vasculocis® reflects a more advanced clinical practice and stronger integration with global supply chains (2). In contrast, countries such as Zambia and Zimbabwe had access to only the basic kits, consistent with findings that procurement barriers limit the expansion of nuclear medicine practice in many Anglophone African countries (13). These results highlight a two-tiered system within SADC, with SA having diversified radionuclide and kit access, global supply links, and local production capacity. Other SADC countries are limited primarily to ^131^I, ^99m^Tc and basic cold kits, with strong dependence on SA or external suppliers. This disparity mirrors the wider African context, where nuclear medicine infrastructure, human resources, and radiopharmaceutical access remain concentrated in a few countries, leaving many with minimal or no services (2, 12, 13).

The varied use of diagnostic and therapeutic radiopharmaceuticals and cold kits across SADC highlights the need for consistent access and reliable supply to ensure that nuclear medicine facilities remain functional (8). A study similarly suggested that the infrequent and inconsistent supply of radiopharmaceuticals limits the development of nuclear medicine in many African countries, as these products form the foundation of clinical practice (12). SA remains the only country in the SADC region with its own means of production and commercializing radiopharmaceuticals. However, studies have shown that English-speaking African countries often express dissatisfaction with SA being their supplier due to its unreliability in product delivery (13).

Economic and logistical constraints strongly influenced supplier choice, with high costs and limited budgets restricting access (13). Transport and customs delays further reduced radionuclide activity and clinical utility (5). Similarly, respondents’ emphasis on the availability and reliability of a supplier reflects the vulnerability of facilities to supply interruptions, a barrier also highlighted in studies on Anglophone African countries (2). The findings of this study revealed that facilities prioritized reliability and availability over regulatory factors, reflecting costs and resource limitations in these settings. In contrast, facilities in Europe and North America prioritize supplier quality, regulatory compliance, and innovation, supported by established frameworks and local production capacity (12, 13).

Due to the limited availability of generators and suppliers, countries have been subject to deliveries restricted to once a week or once a fortnight. This situation is particularly pronounced in low- and middle-income countries, where flight availability have been significantly reduced since the coronavirus pandemic in 2019 (COVID-19) (8) where the variation in lead times is consistent with other findings Participants from Zambia and Zimbabwe reported a lengthy-complicated procurement process which require an approval from authorities outside the health ministry every time an order is placed. This contributes to the delay in the procurement of radiopharmaceuticals (13).

Most English-speaking countries in SADC countries rely on air transport, and a few depend on both air and road transport. The use of road and air transport has been reported in the distribution and logistics of radionuclides in Europe, where road transportation is predominant and mostly used on national levels and occasionally extended to short-distance international distribution. Air transport is the most efficient method when transporting short-lived radionuclides for longer distances (5).

The findings on notification practices are fragmented across the SADC region. In countries such as Tanzania and Mauritius, reliance on the courier services only suggests a more streamlined but potentially vulnerable system, as facilitates depend on a single communication channel. Zambia’s reliance on the sender-only, further emphasizes this risk, as any miscommunication and delays from suppliers could lead to shipment hold-ups or decay of radionuclides. In contrast, Namibia, Zimbabwe, and SA reported using multiple communication channels, which may enhance reliability by providing alternative points of contact, thus reducing the risk of communication breakdowns.

The fragmented communication and notification processes across SADC may contribute to the delays and inefficiencies in the supply chain. In Europe, however, a different model has emerged, where larger radionuclide production facilities frequently subcontract distribution to specialized external companies (5). This approach streamlines logistics, ensuring timely notification and delivery to end-users while reducing the administrative burden on manufacturers. Adopting this model could help address the inefficiencies seen in SADC, where reliance on a single notification source creates supply chain vulnerabilities. For instance, PRISMAP (the European program for isotopes for medical application) provides a coordinated single-entry access system for researchers, hospitals, and pharmaceutical companies to obtain high-purity radionuclides through centralized logistics and standardized procedures. Implementing such a framework in SADC could help standardize communication, improve transparency, and minimize delays (17).

Radiopharmaceuticals are highly time-sensitive, and their clearance through customs is a crucial point in the supply chain. The findings in this study demonstrate inconsistent customs duty exemptions for radiopharmaceuticals across the English-speaking SADC countries. In Zimbabwe, exemptions may be granted for humanitarian imports, provided importers produce supporting documentation from recognized organizations. In Tanzania, duty exemption for some goods exists, including medicine, but whether radiopharmaceuticals consistently qualify remains unclear, as per the findings of this study. In contrast, SA, Namibia, Mauritius, and Zambia generally do not exempt radiopharmaceuticals from customs duties, even though medicines may fall under exempted goods in some countries. This patchwork mirrors the broader supply-chain vulnerabilities described by another study, noting that radioisotopes are often classified as raw materials rather than medical products, leaving them exposed to tariffs and trade barriers (18).

Documentation is a crucial requirement for customs clearance in the radiopharmaceutical supply chain. Importers often face complex documentation procedures, unclear regulations, and lengthy approval times that cause shipment delays and a general lack of clarity regarding customs regulations (19). For radiopharmaceuticals, such delays can result in product decay, directly affecting availability and patient care as demonstrated in the results of this study.

Awareness and expertise among customs personnel were highlighted as determinants of clearance efficiency, and it is recommended to use licensed brokers to improve compliance and reduce errors (19). The findings of this study showed that, in SA, distributors use customs agents that are knowledgeable about radiopharmaceuticals. In addition, there is advocacy for the adoption of customs management software to streamline documentation and tracking (20). This contrasts with a report from a study participant in Mauritius, who indicated that no systems currently in place to improve customs processes, contributing to procedural inefficiencies in customs clearance.

Delays in customs clearance were reported to have consequences, including postponement or cancelation of treatments and production. A study (13) similarly observed that limited awareness among customs and law enforcement officials often leads to clearance delays. Furthermore, in some settings, restrictive security policies such as mandatory escort of radiopharmaceuticals by security forces in Nigeria have further exacerbated these delays.

This study revealed several factors affecting the importation of radiopharmaceuticals, with transportation emerging as a central concern. Challenges included limited international flights, high import costs, and unharmonized regulations. These findings aligned with another study, which reported that sourcing radiopharmaceuticals within Anglophone African countries is often more expensive than procuring from Europe or Turkey. As most SADC countries source primarily from SA, transport costs remain a major barrier, particularly for Namibia, where road transport is the only viable option (13). Importers relying on SA and European suppliers also face restricted flights and unreliable supply chains (2). By contrast, North African countries benefit from regular flight connections to Europe and Turkey, providing a logistical advantage (12).

Similarly, a global study found that certain cold kits, such as ventilation agents for ventilation-perfusion (V/Q) scans, are no longer available, largely due to high import costs, regulatory barriers, limited suppliers, and availability constraints (8). Nonetheless, participants in our study who reported challenges when sourcing internationally still managed to import a range of radiopharmaceuticals and cold kits. This enabled them to perform procedures such as V/Q scans, suggesting that without these systemic importation challenges, access and service delivery could be significantly improved.

Beyond high importation costs, customs duties, and delays, local transport challenges were also found to increase patient care costs and the unavailability of essential radiopharmaceutical kits (8). It has been found that customs processes differ between countries, consistent with this study’s findings (19).

Tanzania was the only participating country where local transport challenges, such as complex regulations, customs clearance delays, and limited or unreliable flights, were specifically reported. In Tanzania, importers are required to obtain appropriate licenses, and the Tanzanian Atomic Energy Commission issues licenses for both customs clearance and the transport of radiopharmaceuticals to nuclear medicine facilities. Participants reported that delays in issuing these licenses restrict transport options and contribute to product decay, highlighting how administrative inefficiencies exacerbate supply chain vulnerabilities (20). Interestingly, while customs duties themselves were not perceived as a major cost driver in this study, respondents stressed that indirect costs arising from delays were significant. Even short delays reduce the activity of radiopharmaceuticals, thereby diminishing their clinical utility and effectively raising the cost of care (8).

In SA, two key challenges were identified, limited number of importers and the cost of shielding. Shielding is necessary to comply with radiation protection standards for storage and transport, but it substantially increases the weight of consignments and therefore air cargo costs (21). Another study also observed that air transport of radioactive sources is constrained by limitations linked to dose rate, shielding, and specialized packaging requirements. These requirements not only raise direct costs but also reduce the number of carriers willing to transport such material (22). In addition, the limited number of companies importing suppliers has created conditions of market monopoly, enabling the control of prices and restricted competition. Dependence on single-source manufacturers or distributors is widespread in nuclear medicine, leaving countries vulnerable to price inflation and supply interruptions (8).

Mauritius reported sourcing difficulties due to its small market size, forcing it to source radiopharmaceuticals from multiple countries. A study highlighted that limited supplier availability and dependency are observed in low-, middle-, and high-income countries globally, underscoring that the issue of constrained supplier markets is a systemic problem rather than one restricted to countries with limited funding (8).

In Tanzania, respondents highlighted high product costs and the absence of local production facilities as their challenges, which made radiopharmaceuticals unaffordable for many patients. This also aligns with the findings of another study, which reported that the limiting factor for importing radiopharmaceuticals is often projected cost, which exceeds allocated budgets (12). Another study also identified high costs of radiopharmaceuticals as a major barrier in Anglophone Africa, emphasizing that costs directly affect patient access (13). Another one suggested that the establishment of local radiopharmaceutical production facilities could improve affordability globally, secure supply, and reduce dependence on imports (8).

A further challenge identified in Tanzania was the lack of government support for radiopharmaceutical services. Policymakers often had limited knowledge of radiopharmaceuticals, and national health priorities favored synthetic drugs and communicable disease programs over cancer and other diseases. Governments across Africa allocate limited budgets to nuclear medicine despite the growing burden of cancer (13). The main barriers to expanding theranostic services in low- and middle-income countries have been found to include restricted access to radiopharmaceuticals and insufficient funding (7).

Furthermore, the study did not include objective indicators such as customs clearance times, transport durations, or cost data, which limits the ability to fully quantify the severity of reported challenges. Although lead-time estimates were collected, these reflect only one dimension of the importation process and do not capture the broader administrative, logistical, or financial burden. Therefore, the severity of these challenges should be interpreted with caution.

The findings of our study revealed several areas where improvements could strengthen the importation of radiopharmaceuticals in the participating SADC countries. Respondents recommended that customs officials receive targeted education on radioisotopes and radiopharmaceuticals to understand the urgency of clearance processes. This highlights the importance of training customs personnel and standardizing documentation to avoid delays that compromise the supply chain of time-sensitive materials (19). Similar calls have been made in previous studies advocating improved inter-agency coordination and awareness in handling radioactive consignments (21). Educating customs officials would therefore not only improve clearance efficiency but also reduce decay-related losses.

High prices and the lack of local production were identified as persistent challenges, with respondents recommending the establishment of local radiopharmaceutical and cold kit manufacturing centers. The expansion of isotope production capacity across Africa has been widely recognized as a sustainable approach to improving access and affordability (7). Developing research reactors through collaboration between governments, universities, and the private sector would enable the local production of key radioisotopes, while local cold kit production could shorten supply chains and stabilize prices. A study similarly proposed that regional production hubs could help reduce the dependency on imports from SA and Europe, particularly for commonly used diagnostic radiopharmaceuticals (13).

Respondents also suggested that radiopharmaceuticals be prioritized in national health policies, with increased funding, subsidies, and inclusion in the National Health Insurance (NHI). This aligns with the recommendations of another study, which found that insufficient budget allocation for radiopharmaceutical services reflects low government prioritization, despite the rising burden of diseases (13). Improved funding mechanisms and inclusion of nuclear medicine in national cancer control strategies are essential for sustainable service provision (7). Strengthening government commitment through policy inclusion and public-private partnerships would therefore address financial barriers and improve access across the region.

The issue of small market size, especially in countries like Mauritius, was discussed in relation to limited supplier interest and high unit costs. Respondents proposed pooled procurement mechanisms, allowing neighboring countries to combine their purchasing power and negotiate better prices. Pooled procurement for small-volume medical commodities is one of the cost-containment and supply-stabilization strategies (23). Such collaboration within SADC could harmonize quality standards and regulatory processes, thereby improving efficiency and supplier confidence.

Addressing market monopolies was another key recommendation. Respondents suggested approving more centers licensed to import and distribute radiopharmaceuticals, which could promote competition and reduce potential price manipulation. Finally, respondents recommended the adoption of digital solutions, such as real-time order systems and shipment tracking tools, to improve transparency and efficiency in the supply chain. Overall, the suggestions provided by respondents emphasize a combination of capacity-building, policy reform, regional collaboration, and technological innovation as key drivers for improving radiopharmaceutical importation and access in SADC countries.

## Conclusion

This pilot, exploratory study assessed barriers to radiopharmaceutical importation across six SADC countries. The importation of radiopharmaceuticals within the English-speaking SADC region faces economic, logistical, and regulatory challenges that impact their accessibility. Addressing these issues requires coordinated strategies such as increasing licensed import centers, exploring local production, fostering government collaboration, investigating pool procurement, educating customs personnel, and encouraging fair regional pricing.

The results provide a foundation for larger-scale studies with expanded respondent recruitment to strengthen generalizability and inform regional strategies aimed at improving radiopharmaceutical supply chains and supporting capacity-building in Africa. Expanding licensed import centers can improve distribution efficiency by decentralizing importation points, thus minimizing transport times and improving timely access (24). Implementing these measures will strengthen nuclear medicine infrastructure and improve patient access to radiopharmaceuticals. Although limited by sample size, the study demonstrates the feasibility of conducting multi-country assessments involving specialized nuclear medicine stakeholders.

## Data Availability

The original contributions presented in the study are included in the article/supplementary material, further inquiries can be directed to the corresponding author.
